# Conservative Treatment for Spontaneous Rectus Sheath Hematoma: A Case Report

**DOI:** 10.7759/cureus.77065

**Published:** 2025-01-07

**Authors:** Hirokazu Matsuura, Masayuki Kojima

**Affiliations:** 1 Department of Surgery, Hitachiomiya Saiseikai Hospital, Ibaraki, JPN

**Keywords:** case report, conservative treatment, emergency medicine, hemostatic agent therapy, spontaneous rectus sheath hematoma

## Abstract

Rectus sheath hematoma is a rare but clinically important cause of acute abdominal pain. In most cases, patients have a history of trauma or are taking antiplatelet or anticoagulant therapy. Spontaneous rectus sheath hematoma is even rarer, with atheromatous changes identified as a contributing factor in the elderly.

A 100-year-old woman with a history of hypertension, chronic obstructive pulmonary disease, and congestive heart failure presented to our emergency department with sudden-onset abdominal pain and a large lower abdominal mass. She was not on anticoagulant treatment and denied any traumatic event or excessive strain on abdominal musculature such as a cough. A CT scan showed a hematoma involving the left anterior abdominal wall at the rectus sheath, extending into the lower abdomen. Subsequently, she was diagnosed with spontaneous rectus sheath hematoma. We started conservative treatment with hemostatic agents, namely, carbazochrome and tranexamic acid to control the expansion of bleeding. Her hemoglobin level and vital signs remained stable, and her symptoms improved significantly. A follow-up CT scan four days after admission revealed a reduction in the size of the hematoma. The patient’s treatment course was uncomplicated, and she was eventually discharged on the sixth day of her admission.

Rectus sheath hematoma is associated with a high morbidity and mortality rate in patients, especially those with additional comorbidities. Early diagnosis and sufficient supportive treatment are crucial for management. We suggest that carbazochrome and tranexamic acid are also effective therapies.

## Introduction

Rectus sheath hematoma (RSH) is a rare but clinically important cause of acute abdominal pain. RSH was first described nearly 2,500 years ago by Hippocrates and Galen [1]. The first case report was reported in 1857 by Richardson [2].

In most cases, patients have a history of trauma, are on antiplatelet or anticoagulant therapy, or have an intense coughing episode. Other risk factors include pregnancy, previous surgery, and various medical conditions such as hypertension or liver cirrhosis. Spontaneous RSH is rarer and more frequent in females and the elderly. Atheromatous changes have been identified as a contributing factor to spontaneous RSH in the elderly [3].

RSH is usually managed with a conservative approach. Invasive intervention is required when conservative management fails, but it is associated with significant morbidity due to advanced age and multiple comorbidities [4].

In this report, we describe a case of a high-risk elderly patient with RSH who was treated with a devised conservative approach.

## Case presentation

**Figure 1:**
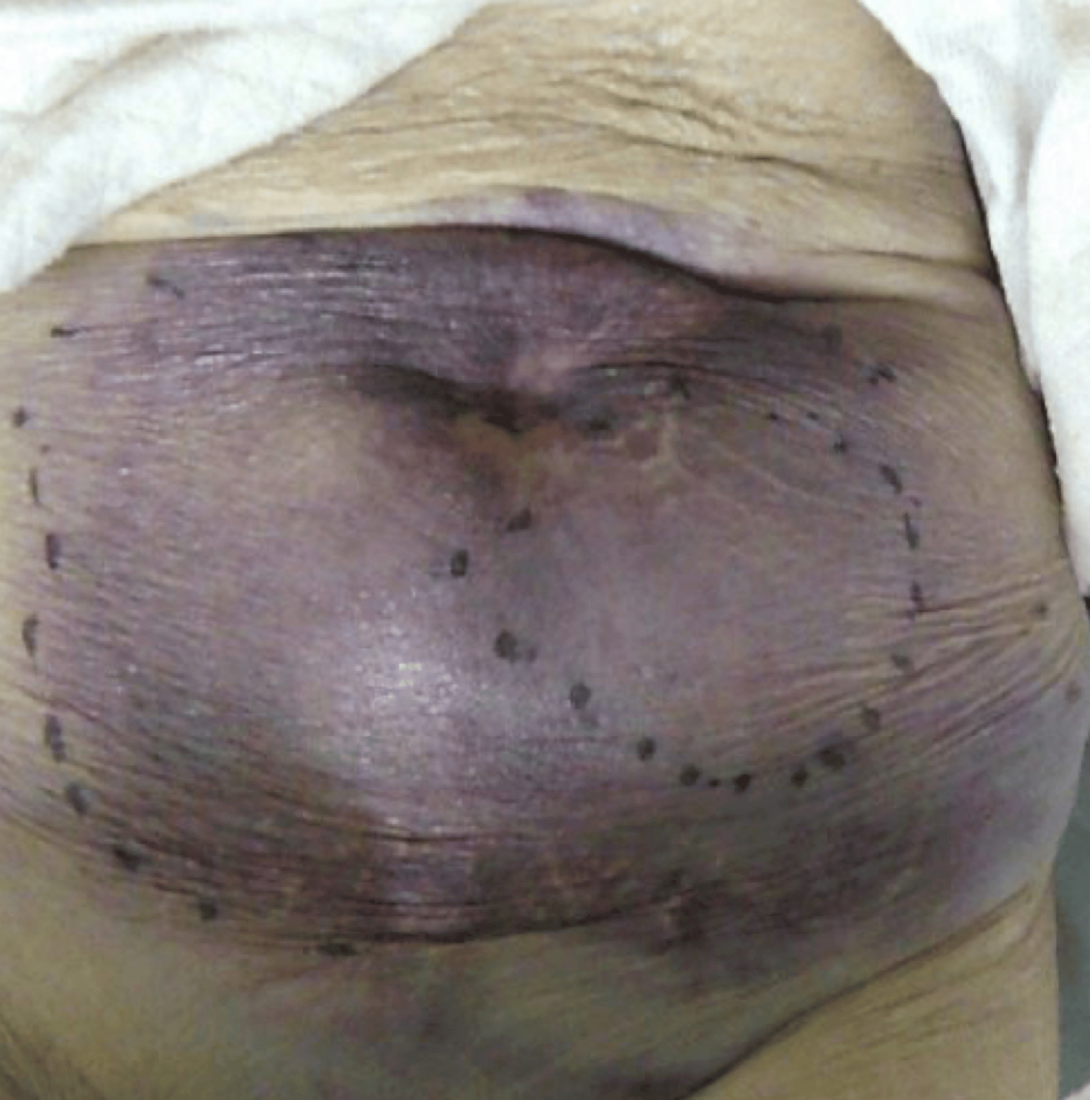
Clinical picture showing an abdominal mass on admission.

A 100-year-old woman with a history of hypertension, chronic obstructive pulmonary disease, and congestive heart failure presented to our emergency department with a sudden onset of abdominal pain and a large lower abdominal mass. She was not on anticoagulant treatment and denied any traumatic events or cough (Figure 1).

A physical examination revealed a hematoma in the infraumbilical region. The mass was tender on palpation, but no peritoneal signs were present. A blood investigation revealed a hemoglobin level of 10.0 g/dL (11.6-14.8 g/dL), platelet count of 79,000/μL (158,000-348,000/μL), activated partial thromboplastin time of 32 seconds (24-34 seconds), prothrombin time-international normalized ratio of 1.03 (0.85-1.25), creatinine of 1.40 mg/dL (0.46-0.79 mg/dL), and estimated glomerular filtration rate 26.5 mL/minute/1.73 m^2^.

**Figure 2:**
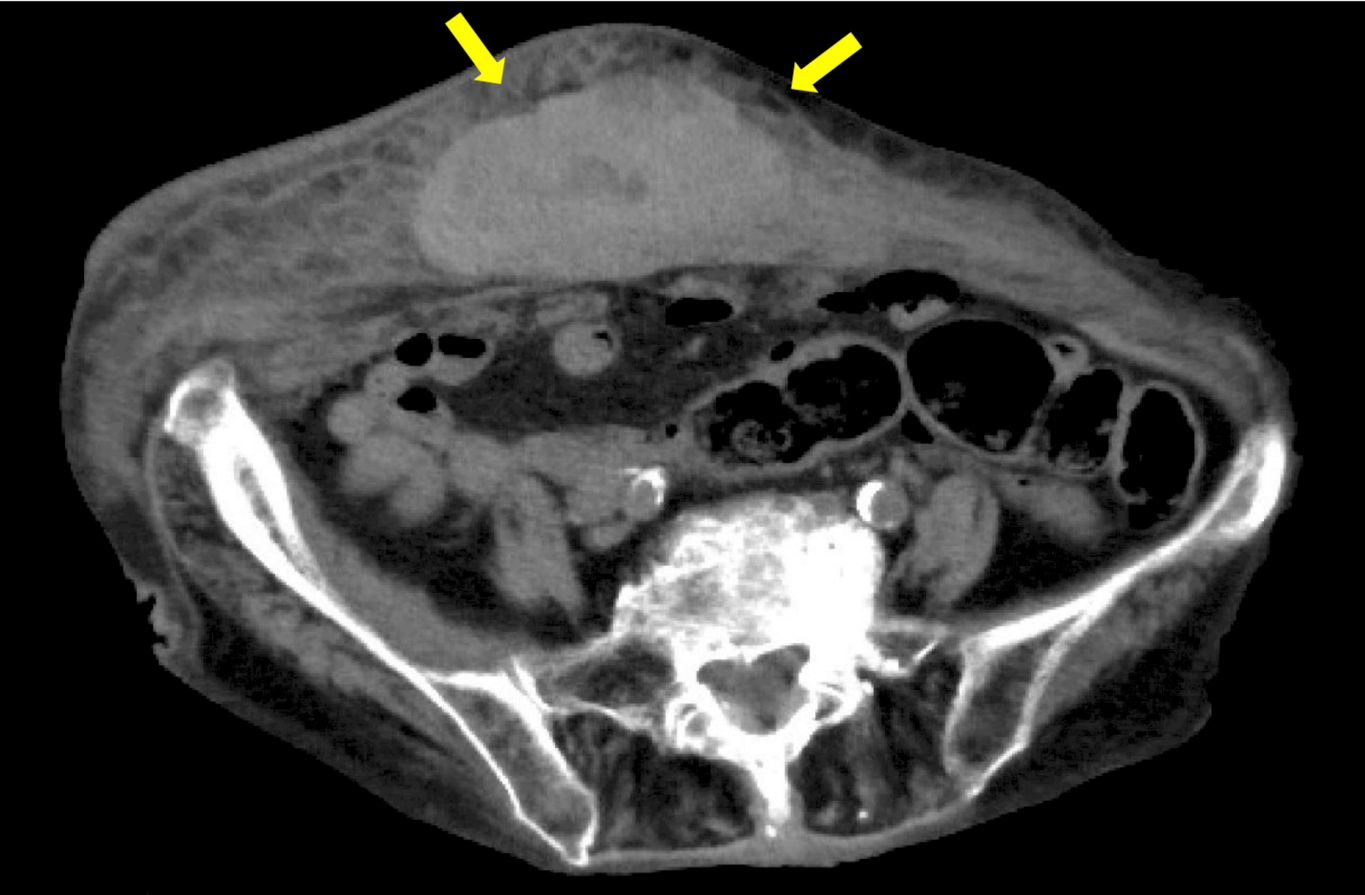
CT scan showing a rectus sheath hematoma on admission.

Suspecting internal hemorrhage, we considered a contrast-enhanced CT of the abdomen and pelvis. Given her impaired renal function, a non-contrast-enhanced abdominal CT was performed. The CT scan showed a hematoma involving the left anterior abdominal wall at the rectus sheath, extending into the lower abdomen. She was diagnosed with spontaneous RSH (Figure 2).

**Figure 3:**
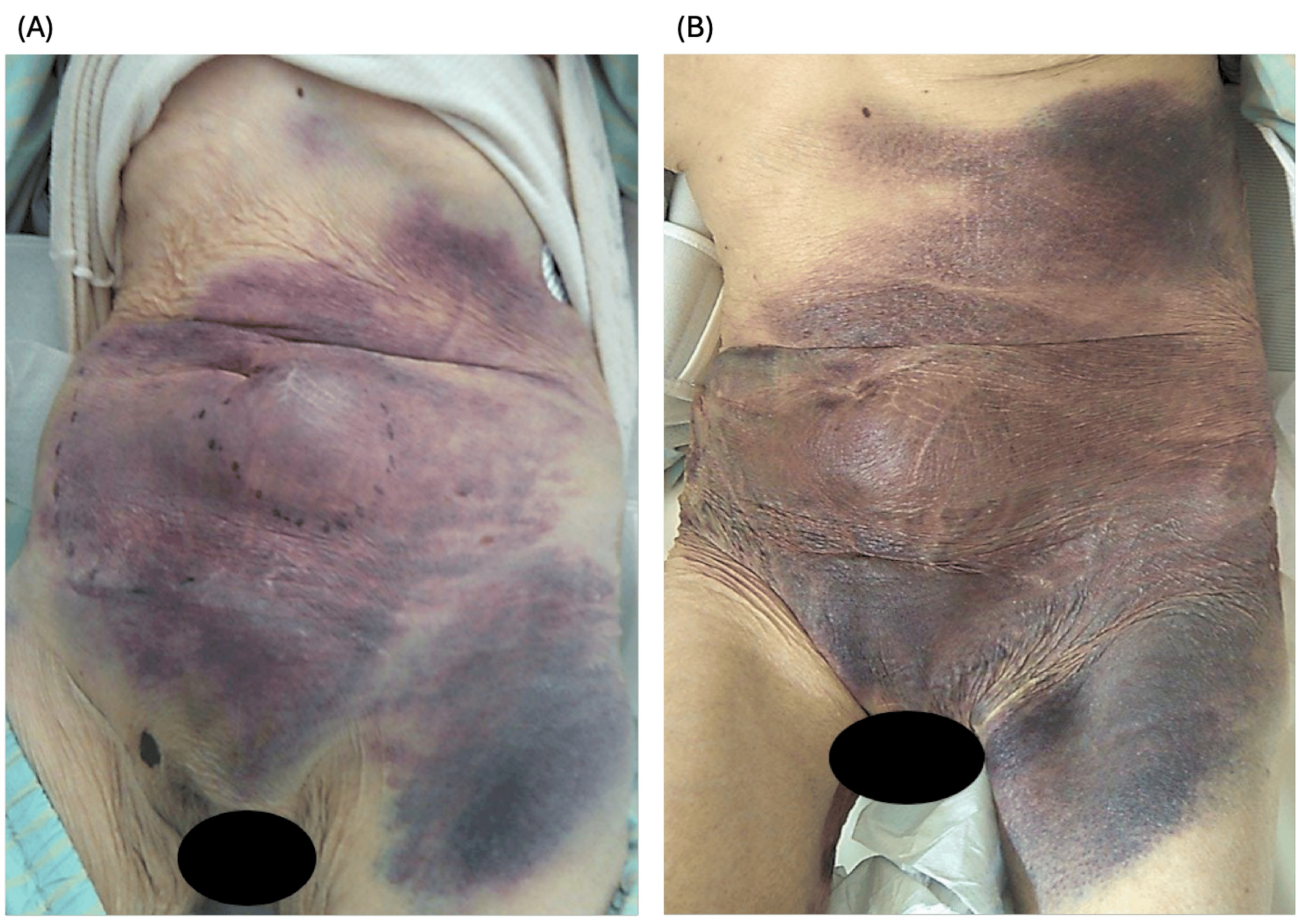
Clinical picture showing an abdominal mass on the second day (A) and the third day (B).

**Figure 4:**
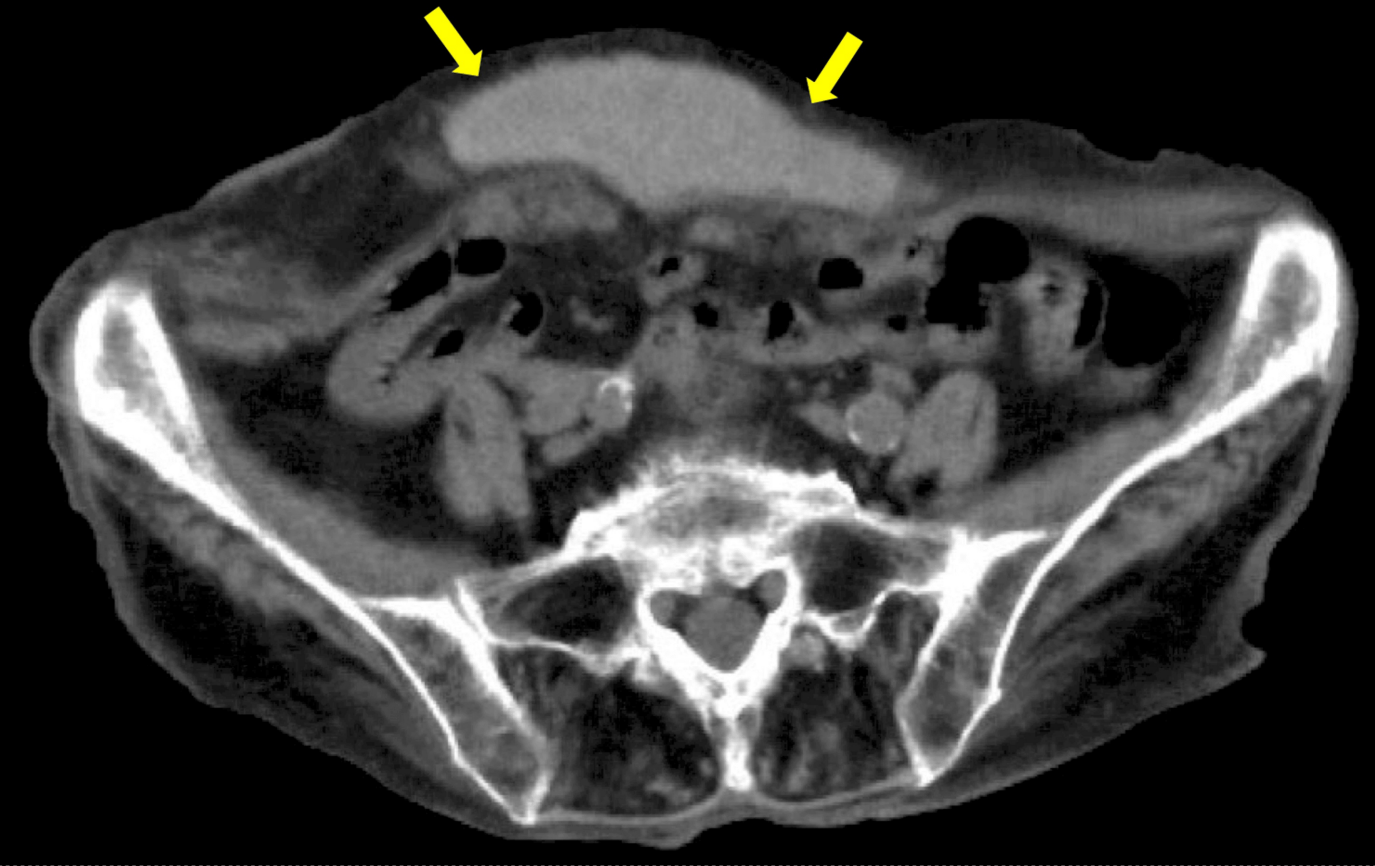
CT scan showing a rectus sheath hematoma on the fifth day.

We started conservative treatment because of the patient’s old age, comorbidities, and moderate frailty, with an interventional approach to be considered if conservative measures were unsuccessful. Furthermore, two hemostatic agents, namely, carbazochrome (50 mg) and tranexamic acid (1,000 mg), were administered intravenously. Additionally, tranexamic acid (1,000 mg) was administered on the first day of admission. Subsequently, tranexamic acid (1,000 mg) was administered twice daily. Her hemoglobin level nadired at 8.0 g/dL two days later and improved gradually afterward. Her vital signs remained stable and her symptoms improved significantly. A follow-up CT scan performed four days after admission revealed a reduction in the size of the hematoma. Consequently, no transfusion and no surgical intervention were required. Her treatment course was uneventful, and she was eventually discharged on the sixth day of her admission (Figures 3, 4).

## Discussion

RSH is defined as the presence of blood within the rectus sheath resulting from the rupture of branches of epigastric vessels. Advanced age and female gender as well as chronic kidney disease and immunosuppressant or steroid therapy are well-known precipitating factors of this uncommon bleeding complication. Anatomically, the inferior epigastric arteries are more prone to injury. RSH is more likely to be observed in the lower than the upper abdominal wall due to the absence of a posterior rectus sheath below the arcuate line. Hematoma above the arcuate line is frequently small and limited between the anterior rectus sheath and muscle fibers. As seen in this case, RSH below the arcuate line, where the rectus abdominis muscle is supported only by transversalis fascia and parietal peritoneum, is characterized by the dissection of tissue planes and the extension across the midline [5].

An accurate diagnosis requires both a high index of clinical suspicion and appropriate radiological investigations. A delay in diagnosis may result in high mortality. As observed in this patient, the most common symptom is a palpable abdominal mass associated with abdominal pain. Upon physical examination, a palpable abdominal mass has been reported in 63-92%, with abdominal guarding in 49% of RSH cases [6].

Tachycardia, diaphoresis, and hypotension are signs of hypovolemia that require urgent medical attention. It is recommended to perform serial hemoglobin and hematocrit measurements and assess the necessity for blood transfusion [1]. Hematocrit levels in RSH patients vary depending on the severity. Furthermore, coagulation factors should be measured to determine the need for reversal in patients on anticoagulation therapy. Fortunately, this case had no signs of hypovolemic shock or coagulation disorder.

CT is an effective diagnostic modality for RSH with nearly 100% sensitivity and specificity [6-8]. It helps detect the location of the hematoma and quantify its expansion and the bleeding source. It assists in planning therapeutic strategies and can be used for the monitoring of hematoma size and activity. The Berna classification, based on the radiographic features on CT, allows for the prognostication of increasing severity. Type I RSH is confined within the rectus muscle without dissecting fascial planes or crossing the midline. Hospitalization is not necessary and the hematoma resorbs spontaneously within 30 days. Type II RSH is also confined within the rectus muscle, but can dissect along the transversalis fascial plane or cross the midline. Type III RSH is usually large with evidence of hemoperitoneum or blood within the prevesical space of Retzius [5,9].

RSH is regarded as a benign condition with a favorable outcome. The overall mortality in RSH is 1.6-5% [10] which increases to 25% in patients undergoing anticoagulation treatment [6,11]. Most cases of RSH are self-limiting and can be managed successfully without the need for invasive surgery. The management of RSH is dependent on the clinical status and classification. A vast majority of RSH patients are treated conservatively, especially with type I and type II RSH. Conservative therapy includes bed rest, pain management, intravenous hydration, blood transfusions, reversing coagulopathy, compression of hematoma, and blood pressure management [5]. Approximately 80% of patients achieve successful outcomes following conservative therapy and treatment of bleeding disorders [12].

In this case, CT revealed the hematoma within the rectus sheath and its extension across the midline which is consistent with a type II diagnosis. Patients with type II and III hematomas are typically hospitalized for treatment, as they require conservative management in our hospital. Our patient was not on anticoagulation therapy and did not have a coagulative disorder, but she was at high risk due to her age. When conservative management fails, invasive intervention is required which can be fatal in such patients. Angioembolization is also associated with complications such as contrast-induced nephropathy. In this case, a surgical approach was associated with a high risk of complications due to the patient’s advanced age and multiple comorbidities. Hyperfibrinolysis may be an emerging plausible pathophysiological mechanism of spontaneous muscle hematomas. To prevent treatment failure, we added hemostatic agent therapy with carbazochrome and tranexamic acid [13]. These agents inhibit the binding of plasminogen to fibrin and reduce the conversion of plasminogen to plasmin. The intervention was efficacious and resulted in favorable outcomes.

## Conclusions

RSH is sometimes associated with morbidity, and invasive intervention therapy also increases morbidity and mortality in the elderly, especially those with additional comorbidities. Early diagnosis and sufficient supportive treatment are essential. In this case, the symptoms improved naturally with conservative management. We suggest that carbazochrome and tranexamic acid are effective therapies.
